# Antibiotic-Loaded Calcium Sulfate as an Adjunct in the Acute Management of a Traumatic Segmental First Metacarpal Defect

**DOI:** 10.7759/cureus.116497

**Published:** 2026-09-18

**Authors:** Álvaro Toro Aguilera, Daniel Grau Checa, Manel Fa Binefa, Lázaro José Vila Lastra

**Affiliations:** 1 Department of Orthopaedic Surgery and Traumatology, Hospital de la Santa Creu i Sant Pau, Barcelona, ESP; 2 Department of Orthopaedics and Trauma Surgery, University of Verona, Verona, ITA

**Keywords:** calcium sulfate, hand injuries, metacarpal bones, open fractures, segmental bone defect

## Abstract

Traumatic segmental bone defects of the hand are uncommon injuries that may require complex reconstruction to restore skeletal stability and function. Calcium sulfate is widely used as a biodegradable bone void filler and local antibiotic carrier, although its use during the acute management of traumatic metacarpal defects has been rarely reported. We present the case of a 55-year-old man who sustained a severe open injury to his dominant hand caused by a radial saw, resulting in dissociation of the first and second rays, segmental bone loss involving approximately 40% of the first metacarpal (approximately 20 mm), and a subcapital fracture of the second metacarpal. Complete laceration of the extensor pollicis longus and flexor pollicis longus tendons was also identified. Following meticulous debridement, microsurgical repair of the deep branch of the palmar arch was performed. The second metacarpal was stabilised with a cannulated screw, while the first metacarpal was stabilised with two Kirschner wires and the osseous defect was filled with gentamicin-loaded calcium sulfate. The injured tendons were subsequently repaired. Complete radiographic union was achieved at three months. At eight months, tip pinch strength was 7 kg and grip strength was 16.2 kg, corresponding to 32% and 45% of the contralateral hand, respectively. The Quick Disabilities of the Arm, Shoulder and Hand (QuickDASH) score was 34. Despite these residual strength deficits, the patient reported satisfaction with the functional outcome and returned to activities of daily living and recreational sports. In this patient, antibiotic-loaded calcium sulfate was used as an adjunctive bone void filler and local antibiotic carrier in the acute setting, when immediate definitive bone reconstruction was not feasible. Further studies are required to determine whether similar outcomes can be reproduced in traumatic metacarpal defects.

## Introduction

Major hand injuries represent some of the most challenging conditions encountered in trauma surgery. These injuries often require rapid decision-making and a multidisciplinary approach to maximize the chances of functional recovery. Chong emphasized that meticulous debridement, skeletal stabilization, restoration of vascularity, and appropriate soft tissue management are essential principles in the management of mangled hands [1].

Evidence-based management recommendations remain limited because severe open hand injuries are relatively uncommon and often involve heterogeneous patterns of bone, tendon, vascular, nerve, and soft tissue injury. Tosti and Eberlin highlighted that initial surgical management is one of the most important factors influencing the success of subsequent reconstructive procedures and overall functional outcome [2].

Segmental metacarpal bone defects present an additional reconstructive challenge. Available treatment options include autologous bone grafting, vascularized bone transfer, and staged reconstruction techniques. Wong et al. reported favorable functional outcomes using a vascularized medial femoral condyle flap for reconstruction of a subtotal first metacarpal defect [3]. However, these procedures may not be feasible during the acute management of severe open injuries. In such situations, simple and readily available adjunctive techniques may be useful while definitive reconstructive options are considered.

Antibiotic-loaded calcium sulfate has been widely used as a biodegradable bone void filler and local antibiotic carrier in the treatment of established bone and joint infections. Oliver et al. demonstrated a favourable biological response to antibiotic-loaded calcium sulfate-based materials, while Hutting et al. and Menon et al. reported its ability to provide high local antibiotic concentrations without requiring a second procedure for removal [4-6]. These reports, however, describe its use in the treatment of confirmed infection; its extrapolation to the acute management of traumatic, non-infected metacarpal bone defects has been scarcely reported.

We present the case of a patient with a severe open hand injury associated with a segmental defect of the first metacarpal that was managed with stable fixation and gentamicin-loaded calcium sulfate. This case highlights a simple and readily available adjunctive approach that may be considered in selected traumatic hand injuries when immediate definitive bone reconstruction is not feasible.

## Case presentation

A 55-year-old right-hand-dominant man presented to the emergency department after sustaining a severe radial saw injury to his dominant hand. Clinical and radiographic evaluation revealed an open fracture of the first metacarpal associated with dissociation of the first and second rays and a segmental bone defect involving approximately 40% of the first metacarpal. A subcapital fracture of the second metacarpal was also identified. In addition, the flexor pollicis longus and extensor pollicis longus tendons were completely lacerated, with an estimated tendon gap of approximately 1 cm.

The patient underwent urgent surgical treatment. Following thorough irrigation and meticulous debridement of all non-viable tissues, microsurgical repair of the deep branch of the palmar arch was performed. Primary repair of the collateral digital nerves was not feasible because of the extent of soft tissue destruction.

The native length of the first metacarpal was estimated from the contralateral hand, measuring approximately 5 cm. Following gentle longitudinal traction, skeletal stabilisation of the first metacarpal was achieved using two 1.5-mm Kirschner wires. Given the traumatic and comminuted nature of the fracture site, a proximal cortical fragment required excision, resulting in an oblique proximal fragment and a segmental defect measuring approximately 20 mm in length - representing approximately 40% of the estimated native bone length. This oblique bone surface precluded stable fixation of a structural bone graft under compression and would have exposed such a graft to shear forces. The defect was therefore filled with a prismatic spacer measuring approximately 20 mm in length and 10 mm in external diameter, prepared using 3 cc of gentamicin-loaded calcium sulfate (Stimulan®, Biocomposites, Keele, UK). The associated second metacarpal fracture was stabilised with a 3.5-mm cannulated screw under fluoroscopic guidance (Figure 1).

**Figure 1:**
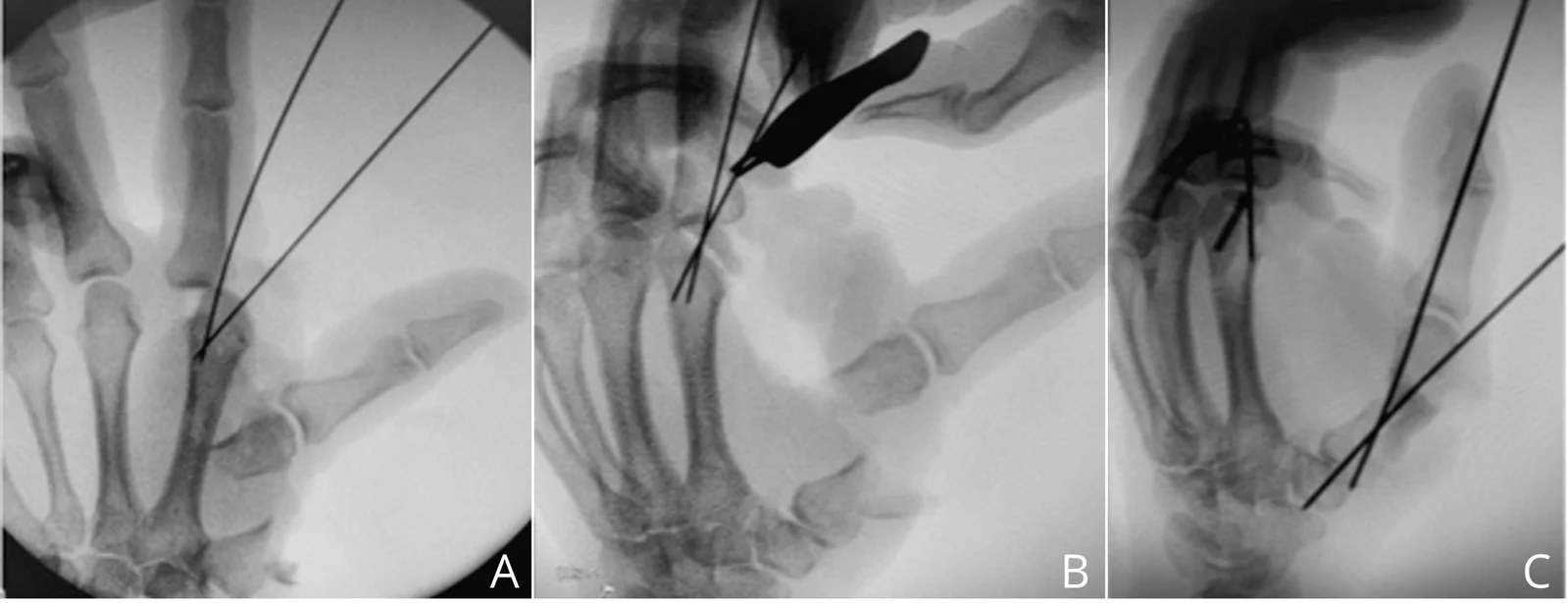
Intraoperative images Intraoperative images showing the traumatic segmental defect of the first metacarpal in panels (A) and (B). Panel (C) shows stabilization of the first metacarpal with two 1.5-mm Kirschner wires and placement of gentamicin-loaded calcium sulfate (Stimulan®, Biocomposites, Keele, UK) within the first metacarpal defect, along with fixation of the second metacarpal using a cannulated screw.

Tendon repair was subsequently completed according to standard surgical principles.

Radiographic union was defined as bridging bone across the segmental defect between the proximal and distal metacarpal segments on at least three of four cortices on orthogonal radiographs, with progressive disappearance of the fracture line and maintenance of alignment. Clinical union was defined by the absence of pain or tenderness at the fracture site and the absence of pathological mobility. Postoperatively, the patient was followed at one, two, and four weeks. The Kirschner wires were removed six weeks after surgery, and progressive radiographic healing was observed. Complete radiographic union of the first metacarpal, as defined above, was achieved at the three-month follow-up (Figure 2). 

**Figure 2:**
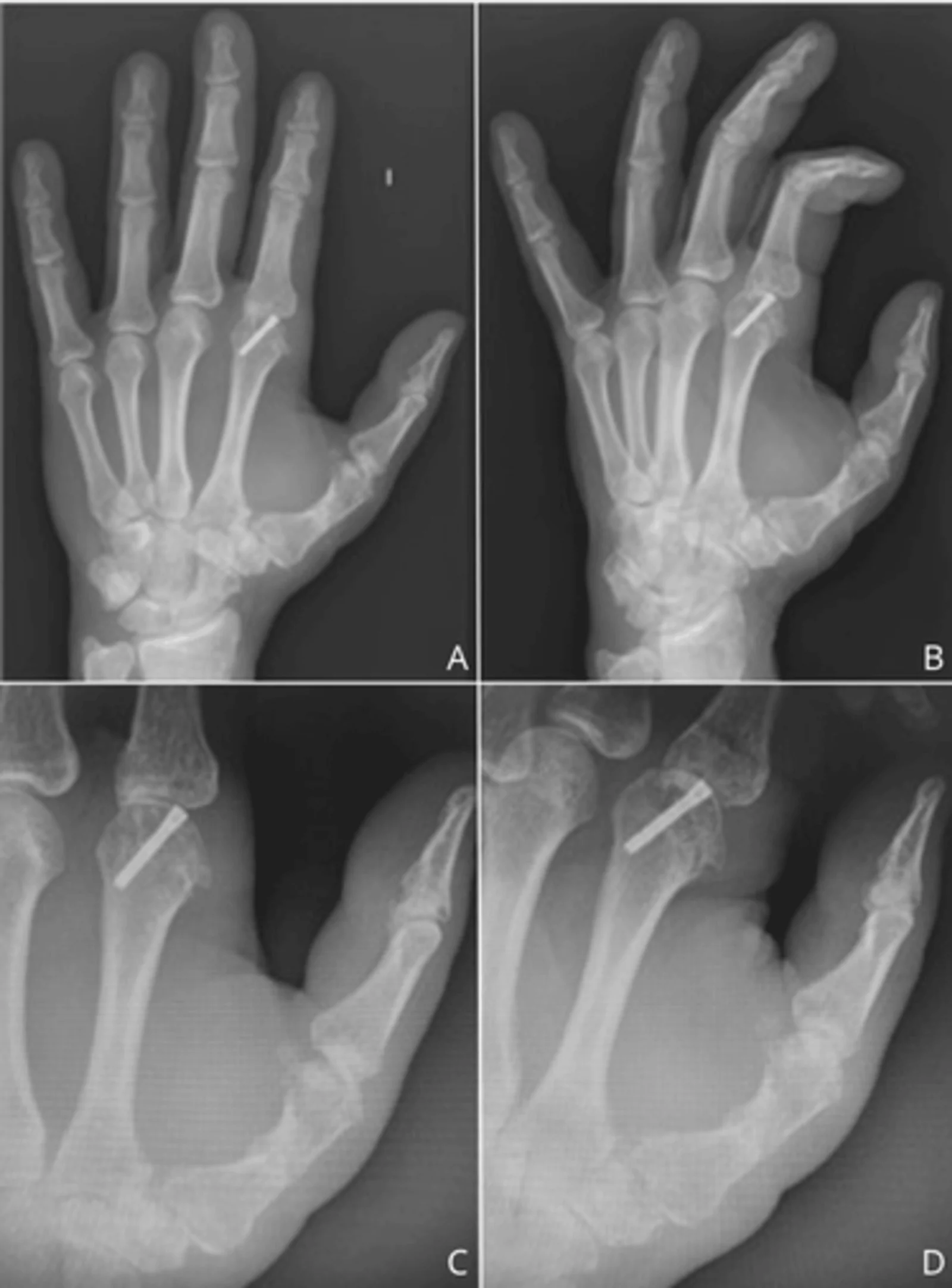
Postoperative radiographs Postoperative radiographs demonstrating progressive bone healing of the first metacarpal. Complete radiographic union was observed at the 3-month follow-up (A, B) and was maintained at the 8-month follow-up (C, D).

No infection developed at any point during follow-up; however, this single observation does not permit conclusions regarding a prophylactic antimicrobial benefit of gentamicin-loaded calcium sulfate. Given the progressive consolidation observed, the second-stage reconstruction initially planned (e.g., Masquelet technique or vascularised bone transfer) was rendered unnecessary.

At the eight-month follow-up, functional assessment demonstrated a tip pinch strength of 7 kg and a grip strength of 16.2 kg, corresponding to 32% and 45% of the contralateral hand, respectively, as measured using a Jamar® dynamometer. Active motion of the index finger was preserved, with 80° of metacarpophalangeal flexion and full extension without pain. The patient achieved a QuickDASH score of 34 [7] and was able to return to activities of daily living and recreational sports. Clinical photographs at the eight-month follow-up demonstrated functional hand use (Figure 3).

**Figure 3:**
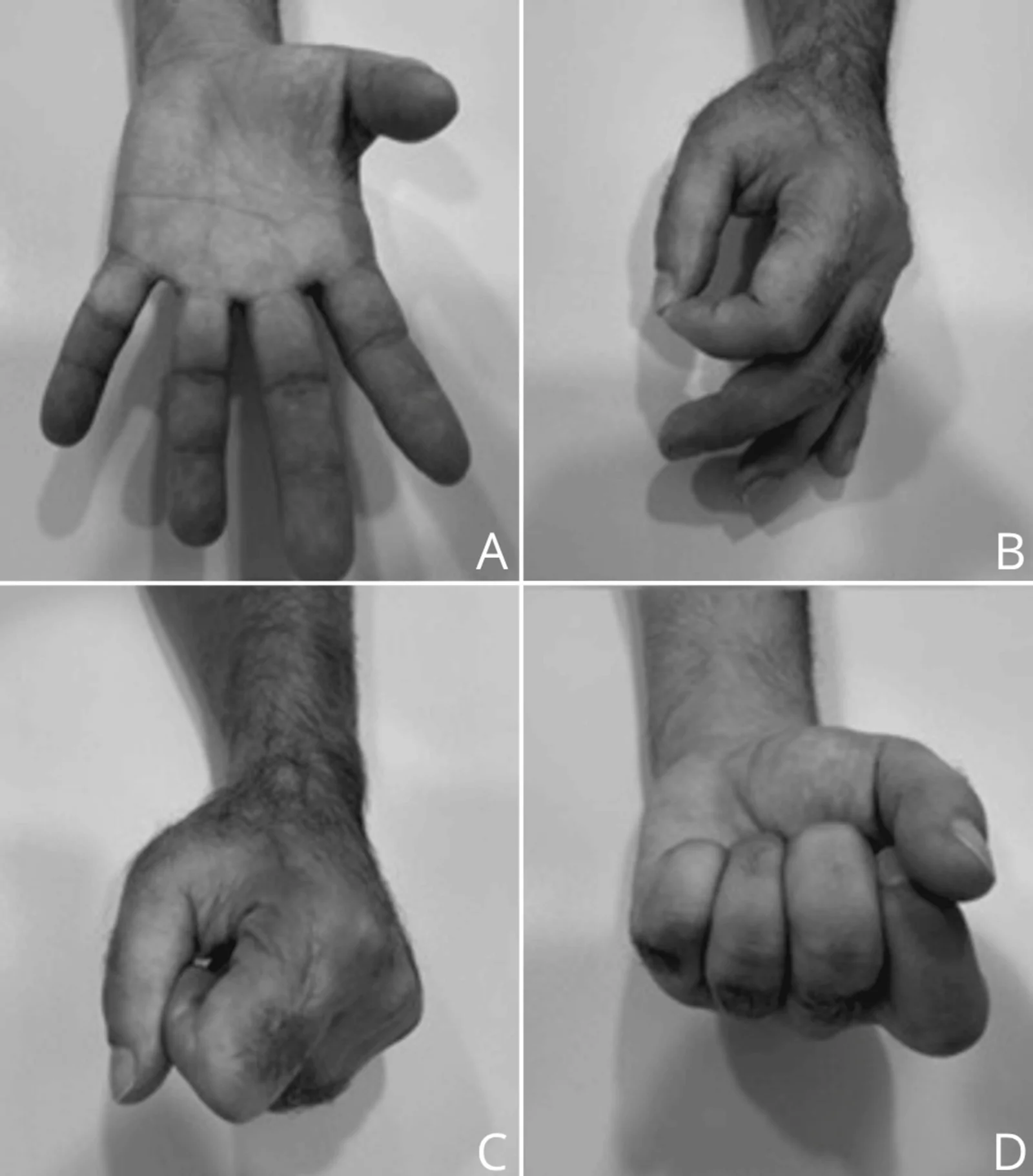
Functional outcome at 8-month follow-up Clinical photographs at the 8-month follow-up demonstrating functional hand use: (A) hand opening; (B) tip-to-tip pinch between the thumb and index finger; and (C, D) fist position.

## Discussion

Major hand injuries remain among the most demanding conditions in trauma surgery because they may involve the simultaneous disruption of bone, tendons, nerves, vessels, and soft tissues. In these situations, treatment priorities extend beyond fracture fixation and include restoration of vascularity, preservation of viable tissue, and planning for functional recovery. As emphasised by Gupta and Wolff and later by Foo and Sebastin, management of these injuries is frequently staged, with secondary reconstructive procedures often required to optimise long-term hand function [8,9].

In the acute setting, thorough debridement and stable skeletal fixation are essential. Depending on the severity of the injury, additional procedures such as pedicled or free flaps, vascularised bone transfer, or toe-to-hand reconstruction may ultimately be required. However, these complex reconstructive techniques are not always appropriate during the initial operation, particularly in contaminated injuries requiring vascular repair and extensive soft tissue reconstruction. In our patient, the immediate priorities were to achieve skeletal stability, restore perfusion, minimise the risk of infection, and preserve thumb length while allowing bone healing to occur. 

Calcium sulfate has gained widespread acceptance as a biodegradable carrier for local antibiotic delivery, particularly in the treatment of established bone and joint infections. Previous experimental and clinical studies have reported favourable biocompatibility and resorption of calcium sulfate, as well as its ability to provide high local antibiotic concentrations without requiring subsequent removal [4-6]. In the present case, however, its purpose was different. Rather than treating an established infection, gentamicin-loaded calcium sulfate was used as an adjunctive bone void filler in the traumatic metacarpal defect, with the additional aim of providing local antibiotic delivery during the early postoperative period. Because calcium sulfate has limited mechanical strength, stable fixation with Kirschner wires remained essential.

In this patient, gentamicin loading was selected because of the contaminated, high-energy mechanism of injury, extensive soft tissue devitalisation, and the need for concomitant microvascular repair, all of which elevate the risk of deep infection even in the absence of a confirmed pre-existing infection. The rationale was therefore prophylactic and adjunctive rather than therapeutic.

The use of antibiotic-loaded calcium sulfate is not without risk. As calcium sulfate resorbs, it characteristically produces a sterile, milky wound exudate that can be difficult to distinguish clinically from a purulent discharge indicative of infection; Menon et al. specifically cautioned that wound status must be interpreted carefully in this context to avoid unnecessary reoperation or antibiotic escalation [6]. Local inflammatory reactions and, less commonly, sterile drainage requiring prolonged wound management have also been reported. Furthermore, the local and systemic pharmacokinetics of gentamicin release in the confined, vascularly compromised environment of a traumatised metacarpal are not well characterised, and supraphysiological local antibiotic concentrations carry a theoretical risk of impairing osteoblastic activity and bone healing. These considerations should be weighed against the theoretical benefits when calcium sulfate is used outside its established indication of confirmed bone or joint infection.

Given a defect length of 20 mm, corresponding to approximately 40% of the native metacarpal length, and an oblique proximal bone surface that precluded stable compression fixation of a structural graft, calcium sulfate alone could not be expected to provide structural support across this gap. This comminuted, oblique fracture margin favoured an approach in which mechanical stability was provided entirely by Kirschner wire fixation, while calcium sulfate served solely as a space-maintaining, antibiotic-eluting void filler rather than a load-bearing construct.

Reconstruction of segmental first metacarpal defects has traditionally relied on autologous or vascularised bone grafts, which provide structural support and reliable biological healing. Wong et al. reported favourable functional outcomes using a vascularised medial femoral condyle flap for subtotal thumb metacarpal reconstruction [3]. Nevertheless, these procedures require microsurgical expertise and are generally reserved for delayed or definitive reconstruction.

Autologous cancellous grafting and vascularised bone transfer were considered but were not appropriate during the index procedure. The oblique morphology of the residual proximal metacarpal fragment did not allow reliable compression and stable fixation of a structural graft. Moreover, the high-energy, contaminated injury and extensive soft-tissue destruction made immediate definitive reconstruction undesirable.

Gentamicin-loaded calcium sulfate was therefore used as a damage-control strategy rather than as definitive treatment, providing temporary management of the segmental defect and local antibiotic delivery while allowing the soft-tissue envelope and vascular status to stabilise. Although a second-stage reconstruction, such as the Masquelet technique or vascularised bone transfer, was initially considered, subsequent radiographs demonstrated progressive bridging and complete consolidation of the defect, making further reconstruction unnecessary.

Calcium sulfate should not be considered a substitute for structural bone grafting in defects requiring mechanical support. Rather, its potential role in this setting is as an adjunctive, biodegradable bone void filler and local antibiotic carrier in carefully selected patients.

To the best of our knowledge, although antibiotic carriers have previously been described for the treatment of bone defects, the literature has predominantly focused on chronic conditions and salvage management of complex infections, with fewer reports addressing their use in acute traumatic metacarpal defects. This report describes the use of antibiotic-loaded calcium sulfate as an adjunct during the acute treatment of a traumatic segmental defect of the first metacarpal. The radiographic outcome observed in this patient cannot be attributed solely to calcium sulfate. Meticulous debridement, stable fixation, vascular repair, tendon reconstruction, and postoperative rehabilitation were all essential components of treatment.

Functional recovery in this patient should also be interpreted in the context of the severity of the original injury. In addition to the segmental bony defect, the patient sustained complete Extensor Pollicis Longus (EPL) and Flexor Pollicis Longus (FPL) lacerations with an approximately 1 cm tendon gap, a vascular injury requiring microsurgical repair, and digital nerve injuries that could not be primarily repaired. Against this background, residual grip strength of 45% and tip pinch strength of 32% of the contralateral hand, together with a QuickDASH score of 34, indicate meaningful but incomplete functional recovery. These outcomes cannot be attributed to bony union alone and reflect the cumulative effect of the combined soft tissue, neurovascular, and skeletal injury and its reconstruction. Nevertheless, the patient subjectively reported satisfaction with the outcome, achieved full independence in activities of daily living, and resumed recreational sporting activities. This divergence between objective strength deficits and patient-perceived outcome has been described elsewhere in the hand surgery literature; Chung et al. found that patient satisfaction following surgical treatment of distal radius fractures correlated only loosely with objective grip strength recovery, with satisfaction thresholds reached well below full restoration of strength relative to the uninjured side [10]. This distinction supports the value of considering patient-reported satisfaction alongside objective strength and validated outcome measures such as the QuickDASH when evaluating recovery after complex hand trauma.

The main limitation of this report is its single-case design, which precludes generalisation of the findings. No conclusions regarding the efficacy of antibiotic-loaded calcium sulfate can be drawn from a single patient. Further clinical studies are required to determine whether this approach provides reproducible benefits in selected traumatic metacarpal defects.

## Conclusions

This case describes the use of gentamicin-loaded calcium sulfate as an adjunctive, damage-control bone void filler and local antibiotic carrier during the acute management of a severe traumatic segmental defect of the first metacarpal, used alongside meticulous debridement, stable skeletal fixation, vascular repair, tendon reconstruction, and rehabilitation. In combination with these measures, the approach was associated with complete radiographic union at three months and a functional outcome - with reduced but improving grip and pinch strength - that allowed the patient to return to activities of daily living and recreational sports, with reported satisfaction.

Calcium sulfate should not be regarded as a demonstrated treatment for segmental metacarpal defects in its own right; rather, this case illustrates one component of a staged, multidisciplinary reconstructive strategy in which definitive bone reconstruction - such as the Masquelet technique or vascularised bone transfer - was initially anticipated but ultimately rendered unnecessary by progressive radiographic consolidation. Its independent contribution to bone healing and infection prevention cannot be determined from a single case, and comparative studies against conventional grafting or staged reconstruction are required before its clinical role can be established.
